# Examining Mental Health, Education, Employment, and Pain in Sickle Cell Disease

**DOI:** 10.1001/jamanetworkopen.2023.14070

**Published:** 2023-05-18

**Authors:** Kelly M. Harris, Liliana Preiss, Taniya Varughese, Anna Bauer, Cecelia L. Calhoun, Marsha Treadwell, Rita Masese, Jane S. Hankins, Faiz Ahmed Hussain, Jeffrey Glassberg, Cathy L. Melvin, Robert Gibson, Allison A. King

**Affiliations:** 1 Program in Occupational Therapy, Washington University in St Louis School of Medicine, St Louis, Missouri; 2 Department of Surgery, Division of Public Health Sciences, Washington University in St Louis School of Medicine, St Louis, Missouri; 3 RTI International, Research Triangle Park, North Carolina; 4 School of Medicine, University of Missouri at Columbia, Columbia; 5 Department of Pediatrics, Pediatric Hematology/Oncology, and Cancer Center, Hematology Program, Yale University School of Medicine, New Haven, Connecticut; 6 School of Medicine, Department of Pediatrics, Division of Hematology, University of California, San Francisco; 7 School of Nursing, Duke University, Durham, North Carolina; 8 Department of Hematology, St Jude Children’s Research Hospital, Memphis, Tennessee; 9 Department of Medicine, Division of Hematology and Oncology, College of Medicine, University of Illinois at Chicago, Chicago; 10 Department of Medicine, Division of Hematology and Medical Oncology, Icahn School of Medicine at Mount Sinai, New York, New York; 11 College of Medicine, Department of Public Health Sciences, Medical University of South Carolina, Charleston; 12 Department of Emergency Medicine, Augusta University, Medical College of Georgia, Augusta; 13 Department of Pediatrics, Division of Pediatric Hematology/Oncology, St Louis Children’s Hospital, Washington University in St Louis, School of Medicine, St Louis, Missouri

## Abstract

**Importance:**

Pain related to sickle cell disease (SCD) is complex and associated with social determinants of health. Emotional and stress-related effects of SCD impact daily quality of life and the frequency and severity of pain.

**Objective:**

To explore the association of educational attainment, employment status, and mental health with pain episode frequency and severity among individuals with SCD.

**Design, Setting, and Participants:**

This is a cross-sectional analysis of patient registry data collected at baseline (2017-2018) from patients treated at 8 sites of the US Sickle Cell Disease Implementation Consortium. Data analysis was performed from September 2020 to March 2022.

**Main Outcomes and Measures:**

Electronic medical record abstraction and a participant survey provided demographic data, mental health diagnosis, and Adult Sickle Cell Quality of Life Measurement Information System pain scores. Multivariable regression was used to examine the associations of education, employment, and mental health with the main outcomes (pain frequency and pain severity).

**Results:**

The study enrolled a total of 2264 participants aged 15 to 45 years (mean [SD] age, 27.9 [7.9] years; 1272 female participants [56.2%]) with SCD. Nearly one-half of the participant sample reported taking daily pain medication (1057 participants [47.0%]) and/or hydroxyurea use (1091 participants [49.2%]), 627 participants (28.0%) received regular blood transfusion, 457 (20.0%) had a depression diagnosis confirmed by medical record abstraction, 1789 (79.8%) reported severe pain (rated most recent pain crises as ≥7 out of 10), and 1078 (47.8%) reported more than 4 pain episodes in the prior 12 months. The mean (SD) pain frequency and severity *t* scores for the sample were 48.6 (11.4) and 50.3 (10.1), respectively. Educational attainment and income were not associated with increased pain frequency or severity. Unemployment (β, 2.13; 95% CI, 0.99 to 3.23; *P* < .001) and female sex (β, 1.78; 95% CI, 0.80 to 2.76; *P* < .001) were associated with increased pain frequency. Age younger than 18 years was inversely associated with pain frequency (β, −5.72; 95% CI, −7.72 to −3.72; *P* < .001) and pain severity (β, 5.10; 95% CI, −6.70 to −3.51; *P* < .001). Depression was associated with increased pain frequency (β, 2.18; 95% CI, 1.04 to 3.31; *P* < .001) but not pain severity. Hydroxyurea use was associated with increased pain severity (β, 1.36; 95% CI, 0.47 to 2.24; *P* = .003), and daily use of pain medication was associated with both increased pain frequency (β, 6.29; 95% CI, 5.28 to 7.31; *P* < .001) and pain severity (β, 2.87; 95% CI, 1.95 to 3.80; *P* < .001).

**Conclusions and Relevance:**

These findings suggest that employment status, sex, age, and depression are associated with pain frequency among patients with SCD. Depression screening for these patients is warranted, especially among those experiencing higher pain frequency and severity. Comprehensive treatment and pain reduction must consider the full experiences of patients with SCD, including impacts on mental health.

## Key Points

**Question:**

What is the association of educational attainment, employment status, and mental health with pain episode frequency and severity among individuals with sickle cell disease (SCD)?

**Findings:**

In this cross-sectional analysis of 2264 individuals with SCD, 47.8% reported frequent pain (ie, ≥ 4 pain crises in 12 months). Although educational attainment and income were not significantly associated with increased pain episode frequency or severity, age, sex, and depression were associated with SCD-related pain.

**Meaning:**

Pain is complex, and these findings suggest that screening patients with SCD for depression and other mental health challenges is warranted, especially among those experiencing higher pain episode frequency and severity.

## Teaser

This cross-sectional study examines the associations of educational attainment, employment status, and mental health with pain episode frequency and severity among individuals with sickle cell disease.

## Introduction

Sickle cell disease (SCD) is a chronic hemolytic anemia that causes organ damage and disproportionately affects individuals of African descent.^1^ Frequent acute vaso-occlusive crises (VOCs) result in chronic inflammation and can lead to acute chest syndrome, severe anemia, and end-organ damage.^2,3,4^ Chronic organ dysfunction is often progressive and experienced by most individuals with SCD at some point throughout the life course.^5,6,7,8^ Complications of SCD can affect educational outcomes and life opportunity for those affected. Cognitive deficits are common and lead to youth with SCD having a lower and often declining intelligence quotient.^9,10^ Lower academic performance, lower test scores, and grade failure are also associated with cognitive deficits.^11,12^ Poor educational outcomes increase students’ risk of dropping out of high school, which, in turn, leads to lower earning potential in adulthood, higher unemployment and incarceration rates, higher poverty, and, ultimately, early death.^13,14,15,16^

Although individuals with SCD experience a variety of complications, pain is a hallmark of the disease and often leads to increased health care utilization and hospitalizations, impacting overall quality of life (QoL).^2^ Pain is complex and does not exist in isolation. SCD-related complications and hospitalizations are associated with social determinants of health, such as socioeconomic status, depression, health literacy, and educational outcomes.^17,18,19,20^ Pain level, frequency, and effect on overall QoL can be impacted by both family-level and neighborhood-level socioeconomic status.^17^ The chronic yet unpredictable nature of the pain and other adverse effects of SCD often leads to increased individual and family stress. These emotional and stress-related effects of the disease may impact daily QoL and frequency and severity of pain.^21^ Mental health is related to opioid use for SCD-related pain and can be seen in stress and negative coping behaviors experienced by individuals with SCD.^22^ Rates of depression among individuals with SCD are approximately 3 times higher than those among the general population (26.0% vs 9.5%),^23,24^ with depressive symptoms impacting pain frequency, health care utilization, health care–related QoL (HRQoL) and stigma (both depression-related and SCD-related stigma). Individuals with SCD, especially young adults, experience greater risk for health-related stigma.^25,26^ Stigma (both perceived and internalized) is associated with disease-related complications of SCD, including pain and health care utilization (ie, frequent emergency department visits and hospitalizations). Specifically, stigma is associated with higher patient-reported disease severity and pain, higher health care utilization, lower QoL, loneliness, and less pain reduction as a result of hospital treatment.^27,28^ SCD-related stigma also has social consequences, including impacts on psychological well-being (including anxiety and depression) or exacerbated pain (perhaps as a result of poor management), and can create challenges in patient-physician relationships.^26^

The pathways through which SCD affects opportunity and life outcomes are relatively clear, but the impact that these outcomes in turn have on SCD symptoms and symptom severity is less defined. Although studies^17,18^ have found that hospital admission frequency may have a limited impact on academic outcomes in youth with SCD, we do know that pain is a factor associated with hospitalizations, stress, and social impacts. Few studies have explicitly examined the associations of SCD-related pain with educational, socioeconomic, and mental health outcomes. To our knowledge, no definitive models exists to clearly define these associations. This study fills this gap because our primary objective was to explore the associations of educational attainment, employment status, and mental health with pain episode frequency and severity in individuals with SCD.

## Methods

### Participants and Study Measures

The Sickle Cell Disease Implementation Consortium (SCDIC) is a National Heart, Lung, and Blood Institute–funded 6-year, 2-phase, multisite, implementation science research study that includes a needs assessment, interventions, and the development of a longitudinal registry of patients with SCD.^29^ Participants for this cross-sectional study were recruited from the 8 clinical centers of the SCDIC: University of Illinois at Chicago (Chicago, Illinois), Duke University (Durham, North Carolina), Washington University School of Medicine (St Louis, Missouri), Mount Sinai School of Medicine (New York, New York), St Jude Children’s Research Hospital (Memphis, Tennessee), Augusta University (Augusta, Georgia), Medical University of South Carolina (Charleston, South Carolina), and UCSF Children’s Hospital Oakland (Oakland, California).^29^ These 8 clinical centers provide services to many of their region’s patients with SCD including those in both urban and rural areas. Participant data from the SCDIC longitudinal registry from all 8 consortium sites were included in the analysis. Registry data included information tracked through the electronic medical record, as well as surveys completed by participants at their medical visit examining the physical, social, and emotional impact SCD has on patients.^30^ Data included in this sample were collected at baseline, from 2017 to 2018. Each participating center obtained institutional review board approval for all study activities and data collection. Informed consent was obtained through signed consent forms. This report follows Strengthening the Reporting of Observational Studies in Epidemiology (STROBE) reporting guidelines for observational studies.

Measures included in the participant survey include the Adult Sickle Cell Quality of Life Measurement Information System (ASCQ-Me), a tool examining HRQoL specifically for patients with SCD.^21,31,32,33^ The ASCQ-Me measures HRQoL in 7 different areas: emotional impact, pain impact, sleep impact, social functioning impact, stiffness impact, pain episodes, and a SCD medical history checklist.^21,32^ Each area is assessed using a 5-item questionnaire, with the exception of the SCD medical history checklist, which uses a 9-item questionnaire. The 5 items examining pain episodes can be used to measure pain episode frequency and severity.^33^ Responses to the first 2 questions, “In the past 12 months how many sickle cell pain attack (crises) did you have?” and “When was your last pain attack (crisis)?” were used to create a composite pain episode frequency score. The following 3 questions—“Using any number from 0-10, where 0 is no pain and 10 is the worst imaginable pain, how severe was your pain during your last pain attack (crisis)?” “How much did your last pain attack (crisis) interfere with your life?” and “About how long did your most recent pain attack (crisis) last?”—were used to create a composite pain episode severity score. Scores from these questions yield a raw score between 0 and 11 for pain episode frequency and 0 and 22 for pain episode severity, which can then be converted to standardized *t* scores for use in the analysis. The *t* scores have a mean of 50 and SD of 10. In the pain episode domain, lower *t* scores indicate lower pain frequency and severity.

Participants provided demographic information, including self-identifying race and sex. Information on race is included as a demographic characteristic but was not included in the analysis. In addition to demographic and disease characteristics (ie, educational and employment characteristics and SCD genotype), the survey examined patient experiences, disease management, and QoL.^30^ Educational attainment response options included less than high school, some high school, high school graduate or general educational development (GED), some college, college graduate, some graduate school, and graduate or professional degree. For the purposes of analysis, high school graduate or GED and some college were combined to create high school graduate, GED, and some college; and some graduate school and graduate or professional degree were combined to create graduate school. Employment status response were categorized as employed, unemployed, or not employed by choice. Medical record abstraction (MRA) was used to identify and merge relevant data elements for participants, including medical diagnoses of anxiety and depression.^30^ Both the MRA data element for diagnosis of depression and patient self-report of current or prior treatment for depression were examined for this analysis. Given similarities in the findings across measures of depression, results of analyses examining self-reported treatment for depression are reported here. It is important to note that while engaged in the study, some community participants had not been seen by a SCD physician as an outpatient in more than 2 years. These participants were unaffiliated with the local site and, thus, did not have MRA data available for inclusion in the analysis.

### Statistical Analysis

 Data analysis was performed from September 2020 to March 2022. Descriptive statistics, including means, SDs, and ranges for continuous variables and frequencies for categorical variables, were used to describe the study sample, patient characteristics, experiences, and HRQoL. Multivariable regression analysis was used to examine the associations of educational attainment, employment status, and mental health with the frequency and severity of pain episodes for individuals with SCD, and to test the hypothesis that, after controlling for age, sex, and treatment (hydroxyurea and pain medication use), individuals with a history of anxiety or depression, lower educational attainment, and periods of unemployment experience more frequent and severe pain crises. In addition to continuous composite measures of pain episode frequency and severity from the ASCQ-Me, separate analyses examining dichotomous variables for pain episode frequency and severity were examined. In the absence of documented guidelines or consistent definitions for more or less severe or frequent pain in SCD, this study used methods previously used by Rizio et al^34^ to determine more severe and less severe pain or frequent pain episodes. Participants were stratified into 2 groups on the basis of pain frequency and severity scores on the ASCQ-Me measures: those with 3 or fewer pain episodes in the prior 12 months (compared with those with 4 or more pain episodes) and those with pain severity scores at least one-half SD above the mean. Multivariable logistic regression was used to examine associations of these categorical variables with educational attainment, employment status, mental health. To ensure best model fit, backward elimination (significance was set at 2-tailed *P* < .05) was used for all regression analyses. All analyses were conducted using SAS software version 9.4 (SAS Institute).

## Results

### Sample Characteristics

A total of 2264 participants from all 8 consortium sites were included in this analysis (Table 1). The mean (SD) participant age was 27.9 (7.9) years (range, 15-45 years); however, nearly 70% of the sample (1541 participants) were aged 18 to 34 years, and less than 10% (209 participants) were younger than 18 years. More than one-half of the sample identified as female (1272 participants [56.2%]), 43.8% (992 participants) identified as male, and 95.5% (2112 participants) identified as Black. Seventy-two percent (1633 participants) had a diagnosis of sickle cell anemia (SCA) which includes the 2 most severe genotypes of SCD (1545 patients [68.3%] with hemoglobin [Hb] SS, the most severe form of SCD, and 88 patients [3.9%] with HbSβ^0^ thalassemia). More than one-half of the participants (1733 participants [78.0%]) reported their highest level of education as a high school diploma or lower. Most were unemployed (1408 participants [63.9%]), although 513 (23.3%) of those who were not employed reported being not employed by choice, and 1083 participants (54.0%) reported an average annual household income less than $25 000.

**Table 1.** Sample Characteristics

Variable	Participants, No. (%) (N = 2264)
Age, mean (SD) [range], y	27.9 (7.9) [15.0-45.0]
<18	210 (9.3)
18-24	641 (28.3)
25-34	900 (39.8)
≥35	513 (22.7)
Sex	
Female	1272 (56.2)
Male	992 (43.8)
Race	
American Indian or Alaska Native	10 (0.5)
Asian	6 (0.3)
Black	2112 (95.5)
Multiracial	76 (3.4)
White	8 (0.4)
Educational attainment	
Less than high school	65 (2.9)
Some high school	334 (15.0)
High school graduate, general educational development, or some college	1334 (60.1)
College graduate	302 (13.6)
Graduate school	185 (8.3)
Employment status	
Employed	796 (36.1)
Unemployed	895 (40.6)
Not employed by choice	513 (23.3)
Annual household income, $	
≤25 000	1083 (54.0)
25 001-50 000	447 (22.3)
50 001-75 000	221 (11.0)
>75 000	254 (12.7)
Disease genotype	
HbSS or sickle cell anemia	1545 (68.3)
HbSβ^0^	88 (3.9)
HbSC	478 (21.1)
HbSβ^+^	126 (5.6)
Other variants	25 (1.1)
Depression diagnosis or treatment	
Self-reported treatment	537 (24.7)
Diagnosis confirmed by medical record abstraction	457 (20.0)
Anxiety	309 (13.7)
Hydroxyurea use (current)	1091 (49.2)
Regular blood transfusions	627 (28.0)
Pain crisis frequency (>4 in last 12 mo)	1078 (47.8)
Pain crisis severity	
Pain rated ≥7	1789 (79.8)
Daily pain medication	1057 (47.0)
Pain crises length (≥4 d)	1114 (49.7)
ASCQ-Me pain frequency score, mean (SD) (n = 2261)	48.6 (11.4)
ASCQ-Me pain severity score, mean (SD) (n = 2259)	50.3 (10.1)

Abbreviations: ASCQ-Me, Adult Sickle Cell Quality of Life Measurement Information System; Hb, hemoglobin.

Nearly one-half of all patients (1057 participants [47.0%]) reported taking pain medication every day for SCD-related pain, and most were taking some form of disease-modifying therapy (hydroxyurea, 1091 participants [49.2%]; regular blood transfusion, 627 participants [28.0%]). One-third of all participants either self-reported or had medical record reports of current or prior treatment for depression (723 participants [33.0%]), and 13.7% had a diagnosis of anxiety noted in their medical record (309 participants). Cross-tabulations examining self-reports of depression (537 participants) and diagnosis of depression in the medical record (457 participants) suggest that one-half of those self-reporting treatment for depression had a diagnosis of depression in the medical record (271 participants [50.5%]), and more than one-half of those with a diagnosis of depression in the medical record also self-reported treatment for depression (271 participants [59.3%]). Mean (SD) pain frequency and severity *t* scores were 48.6 (11.4) and 50.3 (10.1), respectively. A majority of participants (1789 participants [79.8%]) rated their pain as a 7 or higher on a scale of 1 to 10, and nearly one-half of the sample reported more than 4 pain episodes in the prior 12 months (1078 participants [47.8%]) and pain episodes lasting 4 or more days (1114 participants [49.7%]).

### Pain Episode Frequency

Regression results for pain episode frequency can be found in Table 2. The β values indicating the degree of change in the outcome variable are provided, along with 95% CIs and *P* values. In the full model, educational attainment and income were not significantly associated with increased pain episode frequency; however, employment status as unemployed was associated with increased pain episode frequency (β, 2.13; 95% CI, 0.99 to 3.26; *P* < .001). Individuals in the sample who were unemployed had pain episode frequency scores that were, on average, 2.13 points higher than those for employed individuals. Female sex was associated with increased pain episode frequency (β, 1.78; 95% CI, 0.8 to 2.76; *P* < .001), as was age, with all age groups showing lower means than the reference group of 25 to 34 years (age <18 years, β, −5.72; 95% CI, −7.72 to −3.72; *P* < .001; age 18-24 years, β, −1.76; 95% CI, −2.99 to −0.52; *P* = .005; age ≥35 years, β, −2.46; 95% CI, −3.71 to −1.21; *P* < .001). Female participants had pain frequency scores, on average, 1.78 points higher than those for male participants, whereas individuals younger than 18 years, those aged 18 to 24 years, and those aged 35 years and older had lower pain frequency scores than those aged 24 to 35. Controlling for age and sex, self-report of current or prior treatment for depression was associated with increased pain episode frequency (β, 2.18; 95% CI, 1.04 to 3.31; *P* < .001), as was hydroxyurea use (β, 1.15; 95% CI, 0.19 to 2.12; *P* = .02) and daily use of pain medication (β, 6.29; 95% CI, 5.28 to 7.31; *P* < .001). Among those who reported current or prior treatment for depression, those who also reported hydroxyurea use (mean [SE], 1.15 [0.49] points) and daily use of pain medication (mean [SE], 6.29 [0.52] points) had higher pain frequency scores than those who did not report such use. Regression models examining medical record–confirmed diagnosis of depression yielded consistent results for pain frequency (Table 3).

**Table 2.** Adult Sickle Cell Quality of Life Measurement Information System Pain Episode Frequency and Severity^a^

Variable	Pain episode frequency		Pain episode severity	
	Estimate (SE)	*P* value	Estimate (SE)	*P* value
Intercept	44.25 (0.63)	<.001	47.64 (0.58)	<.001
Age group, y				
<18	−5.72 (1.02)	<.001	−5.1 (0.81)	<.001
18-24	−1.76 (0.63)	.005	−1.11 (0.55)	.04
25-34	1 [Reference]	NA	1 [Reference]	NA
≥35	−2.46 (0.64)	<.001	0.23 (0.59)	.40
Female sex	1.78 (0.50)	<.001	2.13 (0.45)	<.001
Employment status				
Employed	1 [Reference]	NA	1 [Reference]	NA
Unemployed	2.13 (0.58)	<.001	1.07 (0.53)	.04
Not employed by choice	−0.18 (0.73)	.80	−0.73 (0.67)	.30
Depression (self-report)^b^	2.18 (0.58)	<.001	NA	NA
Hydroxyurea use	1.15 (0.49)	.02	1.36 (0.45)	.003
Daily pain medication	6.29 (0.52)	<.001	2.87 (0.47)	<.001
Model fit				
*R*^2^	0.17	NA	0.08	NA
Overall model *F*	*F*_9,1839_ = 40.3	<.001	*F*_8,1840_ = 18.9	<.001

Abbreviation: NA, not applicable.

^a^ All models were adjusted for sex and age group.

^b^ Depression was not retained in the final model for pain episode severity.

**Table 3.** Adult Sickle Cell Quality of Life Measurement Information System Pain Episode Frequency for MRA-Confirmed Diagnosis of Depression^a^

Variable	Pain episode frequency	
	Estimate (SE)	*P* value
Intercept	44.26 (0.62)	<.001
Age group, y		
<18	−5.89 (1.02)	<.001
18-24	−1.88 (0.62)	.002
25-34	1 [Reference]	NA
≥35	−2.37 (0.62)	<.001
Female sex	1.90 (0.49)	<.001
Employment status		
Employed	1 [Reference]	NA
Unemployed	2.36 (0.56)	<.001
Not employed by choice	0.06 (0.72)	.90
Depression (MRA confirmed)	2.35 (0.61)	<.001
Hydroxyurea use	1.00 (0.48)	.04
Daily pain medication	6.22 (0.51)	<.001
Model fit		
*R*^2^	0.17	NA
Overall model *F*	*F*_9,1905_ = 41.9	<.001

Abbreviations: MRA, medical record abstraction; NA, not applicable.

^a^ All models were adjusted for sex and age group.

### Pain Episode Severity

Like pain episode frequency, educational attainment and income were not significantly associated with increased pain episode severity (Table 2). Employment status, however, was associated with pain episode severity for individuals who were unemployed (β, 1.07; 95% CI, 0.03 to 2.11; *P* = .04). Individuals in the sample who were unemployed had pain episode severity scores that were, on average, 1.07 points higher than those for individuals who were employed. Age was inversely associated with pain severity for individuals younger than 18 years (β, −5.10; 95% CI, −6.70 to −3.51; *P* < .001) and aged 18 to 24 years (β, −1.11; 95% CI, −2.20 to −0.02; *P* = .04). Individuals younger than 18 years and aged 18 to 24 years had lower pain severity scores (by 5.1 points and 1.11 points, respectively) than individuals aged 24 to 35 years. Female sex was associated with pain severity (β, 2.13; 95% CI, 1.24 to 3.02; *P* < .001). Female participants had pain severity scores, on average, 1.78 points higher than those for male participants. Although depression was not retained in the full models for pain severity, hydroxyurea use (β, 1.36; 95% CI, 0.47 to 2.24; *P* = .003) and daily use of pain medication (β, 2.87; 95% CI, 1.95 to 3.80; *P* < .001) remained associated with increased pain episode severity. Those reporting hydroxyurea use and daily use of pain medication also had higher pain severity scores (mean [SE], 1.36 [0.45] and 2.87 [0.47] points, respectively) than those not reporting such use. Depression was also dropped from final regression models examining medical record–confirmed diagnosis of depression, yielding consistent results.

### More Frequent and More Severe Pain

Logistic regression results examining more frequent pain (≥4 VOCs in 12 months) revealed that age younger than 18 years (odds ratio [OR], 0.50; 95% CI, 0.33-0.76; *P* = .001) and older than 35 years (OR, 0.65; 95% CI, 0.51-0.84; *P* = .001) remained inversely associated with pain frequency, and female sex was associated with more frequent pain (OR, 1.26; 95% CI, 1.03-1.54; *P* = .02). Participants younger than 18 years and older than 35 years were less likely than individuals aged 18 to 35 years to have more than 4 VOCs in 12 months (50% and 65%, respectively), and female participants were just over 25% more likely than male participants to have more than 4 VOCs in 12 months. Controlling for age and sex, employment status as unemployed (OR, 1.66; 95% CI, 1.32-2.08; *P* < .001) and self-reported treatment for depression (OR, 1.55; 95% CI, 1.23-1.95; *P* < .001) were both associated with more frequent pain. Participants who were unemployed or who self-reported treatment for depression were more than 1.5 times more likely than participants who were employed or did not report depression to have more frequent pain (>4 VOCs in 12 months). Individuals taking daily pain medication were 2.56 times more likely than those not taking pain medication to experience more frequent pain (OR, 2.56; 95% CI, 2.10-3.14; *P* < .001). Similarly, age younger than 18 years (OR, 0.56; 95% CI, 0.35-0.86; *P* = .008) and lower annual household income ($25 000-$50 000, OR, 0.73; 95% CI, 0.57-0.95; *P* = .02) were inversely associated with more severe pain, and female sex (OR, 1.44; 95% CI, 1.18-1.76; *P* < .001) and employment status as unemployed (OR, 1.40; 95% CI, 1.11-1.78; *P* = .005) were both associated with more severe pain (Table 4). Younger participants (aged <18 years) and those with lower household incomes ($25 000-$50 000 annually) were less likely than older participants and those with higher incomes to have more severe pain, and female and unemployed participants were 1.5 times more likely than male and employed participants to have more severe pain.

**Table 4.** Logistic Regression Models Examining More Frequent and More Severe Pain^a^

Variable	Probability of ≥4 vaso-occlusive crises in 12 mo		Probability of pain severity score ≥55 (0.5 SD above the mean)	
	OR (95% CI)	*P* value	OR (95% CI)	*P* value
Age group, y				
<18	0.50 (0.33-0.76)	.001	0.56 (0.35-0.86)	.008
18-24	0.93 (0.72-1.19)	.50	1.02 (0.79-1.31)	.90
25-34	1 [Reference]	NA	1 [Reference]	NA
≥35	0.65 (0.51-0.84)	.001	1.09 (0.85-1.41)	.50
Female sex	1.26 (1.03-1.54)	.02	1.44 (1.18-1.76)	<.001
Employment status				
Employed	1 [Reference]	NA	1 [Reference]	NA
Unemployed	1.66 (1.32-2.08)	<.001	1.40 (1.11-1.78)	.005
Not employed by choice	1.04 (0.78-1.38)	.08	1.08 (0.8-1.45)	.60
Annual household income, $^b^				
≤25 000	NA	NA	1 [Reference]	NA
25 001-50 000	NA	NA	0.73 (0.57-0.95)	.02
50 000-75 000	NA	NA	1.11 (0.8-1.54)	.50
>75 000	NA	NA	0.78 (0.57-1.08)	.10
Depression^c^^,^^d^	1.55 (1.23-1.95)	<.001	NA	NA
Daily pain medication^d^	2.56 (2.10-3.14)	<.001	NA	NA

Abbreviations: NA, not applicable; OR, odds ratio.

^a^ All models were adjusted for sex and age group.

^b^ Annual household income was not retained in the final model for more frequent pain.

^c^ Refers to self-reported depression.

^d^ Depression and daily pain medication were not retained in the final model for more severe pain.

## Discussion

The overall findings of this cross-sectional study suggest that, in terms of education and employment, SCD-related pain does not discriminate. It does not matter how educated one is, and neither educational attainment nor income was associated with pain episode frequency or severity. Age and sex were, however, associated with both pain episode frequency and severity, with adolescents and young adults experiencing both less frequent and less severe pain episodes compared with older adults and female participants experiencing more frequent and severe pain episodes compared with male participants. Regardless of indicator (self-report or medical record), depression was significantly associated with pain episode frequency but not pain severity. This finding is particularly important considering the disproportionate impact SCD has on individuals of African descent, the stigma Black youth with SCD encounter when they seek care for VOCs, and the current climate and limitations to treatment for pain.^26^ When stratifying participants with more frequent and more severe pain, regressions yielded consistent results with 2 key differences: employment status as unemployed was associated with both more frequent and more severe pain, and annual household income greater than $25 000 but less than $50 000 was inversely associated with more severe pain.

These findings are consistent with other research^35,36^ in this area demonstrating that intensity of pain and emotion-focused coping were associated with a reduced QoL for youth participants, and that negative feelings including stress were associated with experiences of pain crises among participants. A prior interview study^37^ among youth with SCD and their caregivers examining the impact of life-limiting conditions such as SCD found that pain was described as multidimensional, affecting many facets of participants life, including their psychosocial well-being. Prior studies^36,37^ also found that fear of death and stigma were particularly palpable for youth and young adult participants, particularly during periods of intense pain. Future research to build on this body of work, including continued data collection with a focus on depression symptoms, diagnosis, and treatment, as well as additional analyses examining causation, is warranted.

### Limitations

This study has limitations that should be addressed. Although we cannot make statements of causality given the cross-sectional design of this study, the findings do highlight a consistent association between depression and pain for patients with SCD. This association remains for both patient-reported depression and diagnoses of depression documented in the medical record. Although patient reports of depression are not the best measure, the medical record alone is insufficient. The inconsistencies revealed in these measures suggest that the medical record underreports depression among individuals with SCD. The absence of MRA for unaffiliated or community participants may further suppress the reported prevalence of depression among this population. Taken together, this indicates that our overall understanding, diagnosis, and documentation of depression and treatment for depression are suboptimal.^38^

## Conclusions

The findings of this cross-sectional study suggest that pain interventions cannot ignore screening for depression and other mental health challenges among patients with SCD. Without proper screening and assessments for depression, we might overestimate other factors and overlook key factors or outcomes in this population. Further investigation is needed to both understand depression among this population and its association with SCD-related pain. Better screening for depression and the development of interventions are critical. Pain is complex. We cannot treat SCD-related pain with medications only; rather, we must begin to consider and incorporate holistic or comprehensive approaches to reducing pain. To do so, we must consider the full experiences of patients with SCD.

## Notes

Supplement.
Data Sharing Statement
