# Supplementary material for: Examining Mental Health, Education, Employment, and Pain in Sickle Cell Disease
Source: JAMA Netw Open. 2023 May 18;6(5):e2314070. doi: 10.1001/jamanetworkopen.2023.14070 (PMC10196879; doi:10.1001/jamanetworkopen.2023.14070)
Supplement: Supplement. — Data Sharing Statement [file jamanetwopen-e2314070-s001.pdf]

# Data Sharing Statement

Harris. Examining Mental Health, Education, Employment, and Pain in Sickle Cell Disease. *JAMA Netw Open*. Published May 18, 2023. doi:10.1001/jamanetworkopen.2023.14070

**Data**
**Data available:** Yes
**Data types:** Deidentified participant data
**How to access data:** Data will be available through the NHLBI Data Repository (https://biolincc.nhlbi.nih.gov/home/) in 2023.
**When available:** beginning date: 01-01-2023

**Supporting Documents**
**Document types:** None

**Additional Information**
**Who can access the data:** publicly available
**Types of analyses:** unknown
**Mechanisms of data availability:** publicly available data repository (https://biolincc.nhlbi.nih.gov/home/)
